# Caffeine as a Neoadjuvant Therapy in Parathyroid Adenomas: A Narrative Review

**DOI:** 10.7759/cureus.9958

**Published:** 2020-08-23

**Authors:** Mithun K Reddy, Pooja M Murthy, Sarah M Elsayed, Mir Mehdi Hussain, Chris A Robert

**Affiliations:** 1 Medicine, Vydehi Institute of Medical Sciences and Research Centre, Bangalore, IND; 2 Medicine, Faculty of Medicine, Ain Shams University, Cairo, EGY; 3 Obstetrics & Gynecology, Sunrise Hospital, Pune, IND

**Keywords:** parathyroid gland adenoma, caffeine treatment, hyperparathyroidism treatment, caffeine, parathyroid cancer, primary hyperparathyroidism

## Abstract

Caffeine is the most used central nervous system stimulant drug to date. Many studies have shown the association of caffeine with bone remodeling, urinary calcium excretion, kidney stones, acid peptic disease, and the development of cancer. However, there has been very little research exploring the association between caffeine use and parathyroid gland disorders. We shed light on the possible connection between caffeine and parathyroid adenomas, as suggested in the literature.

## Introduction and background

The first literature introducing caffeine as a medication is “The Canon of Medicine”, written by Avicenna, a Persian physician [1]. Initially, many coffee houses were opened in Saudi Arabia, which was used by many Muslims with an intent to provide energy [1]. For many centuries, caffeine is known to be a central nervous system stimulant [1]. Consumption of coffee has been related to the reduced risk of developing type 2 diabetes mellitus [2]. Like this, many studies have suggested the advantages of coffee consumption to reduce the risk of developing various chronic diseases [2]. Furthermore, coffee intake has been associated with a decreased risk of developing liver damage in people at high risk of various liver diseases [2]. The decrease in the incidence of Parkinson's disease in men and in women who have never used estrogen supplementation after menopause have been associated with regular coffee consumption [2]. The content of caffeine in a cup (150 mL) of homemade coffee varies from 30 mg to 170 mg. Improvement in the performance of athletes in long-duration physical activities was observed in coffee consumption, and, interestingly, for every cup of coffee consumed daily, there was a decrease in the risk of suicide tendency [2]. However, there have been very minimal studies regarding the effect of caffeine on hyperparathyroidism. This is a review of previous literature on how caffeine has an effect on the parathyroid hormone levels and how caffeine can be used as neoadjuvant therapy to prevent the risk of further complications in a hyperparathyroid state.

## Review

Methods

We searched PubMed databases for articles on parathyroid and caffeine. The Medical Subject Headings (MeSH) used to search were “Parathyroid glands/drug effects” (MeSH), “Parathyroid glands/metabolism” (MeSH), “Parathyroid hormone/metabolism” (MeSH), “Parathyroid hormone/genetics” (MeSH), “caffeine/pharmacology” (MeSH), “caffeine/administration and dosage” (MeSH), “Parathyroid hormone/blood” (MeSH), “disease models/animals” (MeSH), ”parathyroid gland/ultrastructure” (MeSH). Terms other than MeSH terms used were caffeine and parathyroid gland, caffeine, parathyroid hormone (PTH), parathyroid gland, hyperparathyroidism, parathyroid adenomas, diet, and parathyroid adenoma and caffeine and primary hyperparathyroidism. All human and animal study articles were included, and there are no restrictions on the date of publications. Based on the title and abstract, we searched for our results. We used a citation-based search strategy in which we reviewed citations and references for other relevant articles.

Discussion

Caffeine

Caffeine is found in espresso beans, tea leaves, and cocoa beans. It is regularly ingested as beverages and hence has become the most commonly ingested pharmacologically active substance around the globe [3]. It passes through the gastrointestinal tract rapidly and gets completely absorbed into the bloodstream [3]. It gets metabolized in the liver and has a plasma half-life of approximately five hours [4]. Its elimination half-life ranges from 1.5 to 9.5 hours [4]. Highest plasma concentrations are observed after 15-120 minutes after intake, and the reason for this range of time is the existence of other constituents that are co-ingested with caffeine and different time of gastric emptying [4]. The estimated total plasma clearance rate is 0.078 L/h/kg [4]. The fatal dose of caffeine is 150-200 mg/kg body weight [4]. However, intake of doses up to 10g has resulted in convulsions and emesis, and full recovery is possible within 6 hours [4]. Caffeine acts on the skeletal muscles and adipose tissue enzymes by increasing the cAMP (cyclic adenosine monophosphate) intracellular concentrations, which, in turn, inhibits the phosphodiesterase enzymes [4].

Its adverse effects include rapid bone loss at the spine in postmenopausal females ingesting more than 300 mg/day, especially putting women with TT genetic variant of calcitriol receptor at a higher risk [5]. Caffeine declines bone mineral density, increases the risk of hip fracture, and contrarily impacts calcium maintenance [5]. Caffeine-containing beverage consumption is associated with diminished bone mass and increased fracture risk in high-risk populations [5]. It is well tolerated on regular intake, and though it might affect the stimulant properties, it does not affect the fat cell lipolysis [5]. On an abrupt stoppage of caffeine consumption, withdrawal symptoms such as nervousness, headache, testiness, and reduced energy levels are seen [6]. Oral doses of caffeine can build the urinary excretion of calcium, sodium, chloride, and magnesium for a minimum of 3 hours after the intake, and the dangers of osteoporosis-related with caffeine utilization are investigated [7]. Higher calcium absorption from the intestine remunerates for intestinal and urinary losses [7]. However, elder women do not appear to remunerate sufficiently to keep up their previous calcium balance, particularly in cases when the intake of calcium is below recommendations [7]. Secondary hyperparathyroidism is a common complication of chronic kidney disease and is usually characterized by elevated PTH levels secondary to changes in the homeostasis of calcium, phosphate, and vitamin D [8].

Hyperparathyroidism and Parathyroid Adenoma

Autonomous production of PTH is the characteristic of primary hyperparathyroidism and this has been associated with various malignancies, as well as with premature death in malignant disorders [9]. With a prevalence of 0.1-1.0%, hyperparathyroidism is the third most common clinical endocrine disorder following diabetes and thyroid disorder and is associated with an incidence of approximately 28 cases per 100,000 individuals in the general population [10-12]. Hyperparathyroidism affects 2% of the population aged 55 years or older and its incidence is higher in women more than men, the highest between 50 and 60 years of age [11]. The prevalence of primary hyperparathyroidism has been estimated to be as high as 21 in 1,000 in postmenopausal women [13]. Parathyroid tumors are classified into an adenoma, hyperplasia, cystic changes, and carcinoma [14]. Compared to hyperplasia, parathyroid adenomas often present with a higher increase in the level of serum PTH [14]. Parathyroid adenoma is part of a range of parathyroid proliferative disorders, which include parathyroid hyperplasia, parathyroid adenoma, and parathyroid carcinoma [15]. Parathyroid adenoma is responsible for 80% to 85% of primary hyperparathyroidism cases followed by primary parathyroid hyperplasia (15%) and parathyroid carcinoma (5%) [15]. Parathyroidectomy is the only cure for parathyroid adenomas, which is successful about 95% of the time [16].

The possible effect of caffeine on hyperparathyroidism: evidence

Human beings and hamsters have parathyroid glands that develop from the third and fourth branchial pouches [17]. Hamsters have only two parathyroid glands, but humans have four glands in close proximity to the thyroid gland [17]. Under a light microscope, the parenchyma of the parathyroid gland is made of basal cells and chief cells, which are suprabasal and oxyphil cells [17]. Under an electron microscope, there might be a slight variation in the structure that is considered an artifact resulting from tissue preparation [17]. Accordingly, the parathyroid glands in humans and hamsters are similar in microscopic structure and function [17]. A study was conducted on 42 adult female hamsters of average body weight (135 gm) to find a correlation between the administration of caffeine and any changes in the function and structure of the parathyroid glands and if that affects the bone metabolism [18]. The 42 hamsters were divided into two groups (21 hamsters in each) for 17 and 32 days, respectively. Each group was eventually split into three groups of seven hamsters. One group was the control group that received 0.5 mL of distilled water for each 100 gm body weight. A second group received 2.5 mg of caffeine dissolved in 0.5 mL water for each 100 gm body weight equivalent to 340 mg/70 kg in human bodies. The third group received 10 mg of caffeine dissolved in 0.5 mL water for each 100 gm body weight equivalent to 1,360 mg of caffeine in a 70 kg person [18]. All the doses were given orally once daily. Serum calcium was measured and transmission electron microscopy of the parathyroid gland was performed on day 18 for the first group and on day 33 for the second one. Bodyweight, mineral content of bone (bone mineral density/body weight), and bone mineral density were measured for all groups on day 0 before the study started and on the last day for each group [18]. The results showed no significant difference in serum calcium levels for all groups. When examined the parathyroid glands, there was a remarkable increase in the number of mitochondria, the surface area of rough endoplasmic reticulum, and the Golgi apparatus in the caffeinated groups, suggesting increased PTH synthesis. Measuring the PTH was not possible in that study [18]. As regards to body weight and bone mineral density, there was no significant difference for the 17-day group. For the 32-day group, despite an increase in both weight and mineral density of bone, it was slower in the caffeinated group than the control, and the mineral content of bone was decreased in the highly caffeinated group. This suggests that caffeine affects body metabolism but not necessarily bone mineral density or content [18].

A study was conducted of 25 patients, of whom 19 were females and 6 were males [19]. The patient's age was between 42 and 87 years and their average age was 57 years. The PTH range was 69-1,163 ng/L, with an average serum PTH levels of 361 ng/L, and average serum ionized calcium levels were 1.46 mM, with a range of 1.38-1.59 mM [19]. Of the 25 patients, 22 had primary parathyroid adenoma, 2 had lithium-induced hyperplasia or adenoma, and 1 had secondary parathyroid hyperplasia [19]. The likely effects of caffeine on PTH release were determined by the batch incubation of the patient’s parathyroid adenoma cells [19]. Physiological concentrations of caffeine range from 1 to 50 μM. Caffeine of physiologically significant concentrations of 1 μM, 10 μM, and 50 μM was assessed. Caffeine treatment of a duration of 30 minutes was applied on each plate [19]. It was observed that 50-μM caffeine gave rise to a remarkable reduction in secretions of PTH and expression of the PTH gene [19]. There was an additional associated decline of the activity of protein kinase A, intracellular cAMP level, expression of adenosine receptor (ADOR) A1 gene, showing a potential causal relationship. It was observed that intracellular caffeine levels are not affected by caffeine as it did not show any change even at greater concentrations [19]. ADORA1 and ADORA2A were targeted by caffeine as detected by protein expression observed in the western blot examination [19]. Thirty minutes after the caffeine treatment of adenoma cells of the parathyroid, expression of ADORA2A, ADORA1, and PTH expression was judged by qRT-PCR [19]. A noteworthy decline of 15.3% of PTH mRNA was observed after adding 50-μM caffeine, yet not at 1 μM or 10 μM. On treatment with 50-μM caffeine, PTH secretion was significantly inhibited by 10.4% [19]. For comparison, 0.5-mM calcium caused a 12.4% increase, whereas 1.8-mM calcium led to a 30.5% reduction in the PTH release [19]. A high caffeine dosage inhibits PTH secretion, which might be because of a reduction in the intracellular cAMP levels [19].

This research, however, is subject to several limitations. The first is that there has been very little research on the effect of caffeine on parathyroid glands. The second concerns very little sample data on this topic, and the third concerns the level of the PTH, which was not measured in the trial with the hamsters and was assumed based on microscopic changes in the parathyroid gland and bone mineral density. Therefore, we need more studies on this topic to support the available evidence.

## Conclusions

Very less is understood regarding the impact of caffeine on PTH and its use in hyperparathyroidism. Based on the data reviewed, it is evident that caffeine can be used as adjuvant therapy in parathyroid adenomas to suppress the PTH level until parathyroidectomy is performed. Caffeine too has side effects as mentioned previously if used injudiciously. To prevent such complications, we need additional research analysis on the dosage of caffeine necessary to safely suppress PTH. More research is needed on the effect of caffeine as adjuvant therapy of parathyroid adenomas.
